# Thermal Behavior and Structural Study of SiO_2_/Poly(ε-caprolactone) Hybrids Synthesized via Sol-Gel Method

**DOI:** 10.3390/ma11020275

**Published:** 2018-02-10

**Authors:** Stefano Vecchio Ciprioti, Riccardo Tuffi, Alessandro Dell’Era, Francesco Dal Poggetto, Flavia Bollino

**Affiliations:** 1 Department of Basic and Applied Science for Engineering (S.B.A.I.), Sapienza University of Rome, via del Castro Laurenziano 7, Roma, I-00161, Italy; alessandro.dellera@uniroma1.it; 2 Department of Sustainability, ENEA-Casaccia Research Center, Via Anguillarese 301, Rome, 00123, Italy; riccardo.tuffi@enea.it; 3 Ecoricerche S.r.l., Via Principi Normanni, Capua 81043, Italy; amm.ecoricerche@virgilio.it; 4 Department of Industrial and Information Engineering, University of Campania Luigi Vanvitelli, via Roma 29, Aversa, 81031, Italy

**Keywords:** sol-gel method, SiO_2_–based hybrids, poly(ε-caprolactone), TG-DSC, TG-FTIR, X-ray diffraction analysis

## Abstract

SiO_2_-based organic-inorganic hybrids (OIHs) are versatile materials whose properties may change significantly because of their thermal treatment. In fact, after their preparation at low temperature by the sol-gel method, they still have reactive silanol groups due to incomplete condensation reactions that can be removed by accelerating these processes upon heating them in controlled experimental conditions. In this study, the thermal behavior of pure SiO_2_ and four SiO_2_-based OIHs containing increasing amount (6, 12, 24 and 50 wt %) of poly(ε-caprolactone) (PCL) has been studied by simultaneous thermogravimetry (TG) and differential scanning calorimetry (DSC). The FTIR analysis of the gas mixture evolved at defined temperatures from the samples submitted to the TG experiments identified the mechanisms of thermally activated processes occurring upon heating. In particular, all samples already release ethanol at low temperature. Moreover, thermal degradation of PCL takes place in the richest-PCL sample, leading to 5-hexenoic acid, H_2_O, CO_2_, CO and ε-caprolactone. After the samples’ treatment at 450, 600 and 1000 °C, the X-ray diffraction (XRD) spectra revealed that they were still amorphous, while the presence of cristobalite is found in the richest-PCL material.

## 1. Introduction

In recent years, organic–inorganic hybrids (OIHs) have played a crucial role in the development of multifunctional nanostructured materials [1,2,3,4,5,6,7]. OIHs are not a simple physical mixture of organic and inorganic phases possessing properties that are the sum of those of both components; rather, they are intimately mixed, with average dimensions ranging from a few Å to several nanometers [8,9] These materials have been divided into two classes according to the nature of the bonds between them [10]. Class I consists of those forming weak hydrogen bonds or van der Waals forces, and Class II contains materials obtained by strong chemical bonds (covalent or ionic covalent bonds) between the components. As a matter of fact, research in this area is supported by the growing interest of all materials scientists who are looking to fully exploit this opportunity for creating smart materials that benefit from synergetic or complementary effects exerted by the two phases embedded in one [8,10].

A wide versatility in the design of OIHs may be achieved if they are synthesized at low temperature. In this regard, the sol-gel technique has several advantages over other synthesis processes: it is versatile, since glasses and ceramics may be produced at low temperatures. The transition of the system from a colloidal liquid (‘sol’) into a solid ‘gel’ occurs via hydrolysis of a metal alkoxide precursor and polycondensation reactions occurring in a water-alcohol solution [11,12]. After drying of the obtained ‘wet gel’, and depending on the heat treatment carried out on the ‘dry gel’ it is possible to obtain several products, such as xerogel, areogel or dense ceramics (by a sintering process of the xerogel or the aerogel). The starting low temperature condition allows the chemical homogeneity of the various elements to be controlled down to the atomic level, and thermolabile molecules (e.g., polymers and drugs) to be entrapped in the inorganic matrix, thus producing OIHs [8,13].

Recently, silica-based organic-inorganic hybrids have been attracting the growing interest of several research groups, leading to the development of functional materials for many application areas [14,15,16,17,18]. In the last two–three years, our group has also been involved in preparing (via the sol-gel method) and characterizing SiO_2_-based glasses, ceramics [19,20,21,22,23], as well as SiO_2_-based OIHs, with particular reference to SiO_2_/polyethylene glycol (SiO_2_/PEG) hybrids containing increasing percentages of PEG (from 6 to 70 wt %) [24,25]. More recently, SiO_2_/PCL hybrids containing variable percentages of PCL (6, 12, 24 and 50 wt %) were synthesized via sol-gel and characterized by means of several instrumental techniques [26]. FT-IR, NMR, XRD and SEM analyses showed that the SiO_2_/PCL materials were amorphous and homogeneous organic-inorganic hybrid materials in which the C=O groups in the PCL chains form H-bonds with the –OH groups of the silica matrix. Some studies reported in the literature proved that those hybrids were bioactive and biocompatible [27,28,29]. For this reason, their use was proposed in the biomedical field to prepare coatings able to enhance biological performances of metallic implants [30] or as drug delivery matrices [2].

Some studies reported in the literature showed that the biological properties of the sol-gel materials were affected by the heat treatment carried out after the gel formation [31,32,33,34,35]. When the transition from sol to gel occurs, indeed, both hydrolysis and condensation reactions are incomplete and, thus, reactive silanols are still present in the system. A heating treatment at relatively high temperatures (100–600 °C) is necessary to accelerate this phase, thus removing the organic species and leading to formation of covalent Si-O-Si bonds [11]. Therefore, heating temperature and rate can affect microstructure and crystallization degree of the final material, influencing, in turn, the ion release from the materials [36]. This property is a key factor in determining material biological characteristics, because it can cause modification in the material surface charge and, thus, in protein adsorption [37] and hydroxyapatite nucleation [38].

The aim of the present investigation has been, therefore, to examine and closely compare the thermal behavior of pure SiO_2_ (denoted with S) with that of four SiO_2_/PCL hybrids containing 6, 12, 24 and 50 wt % of PCL (with the symbols SP6, SP12, SP24 and SP50, respectively). The focus of the present study has been to determine the mechanisms of reactions occurring in these materials upon heating them under inert atmosphere by coupling TG and FTIR devices, similar to what has previously been done using TG and mass spectrometry [39]. Such information associated to specific biological studies could allow the rational fine tuning of biomaterials with properties (e.g., crystallization degree, ion release ability, protein adsorption ability, osseointegration ability, etc. [31,32,36,37,38]) adequate to specific applications. To this end, it is also useful to detect by XRD the modification of the solid phases induced upon heating and stables at these temperatures.

## 2. Results and Discussion

### 2.1. Thermal Behavior Study

**Figure 1:**
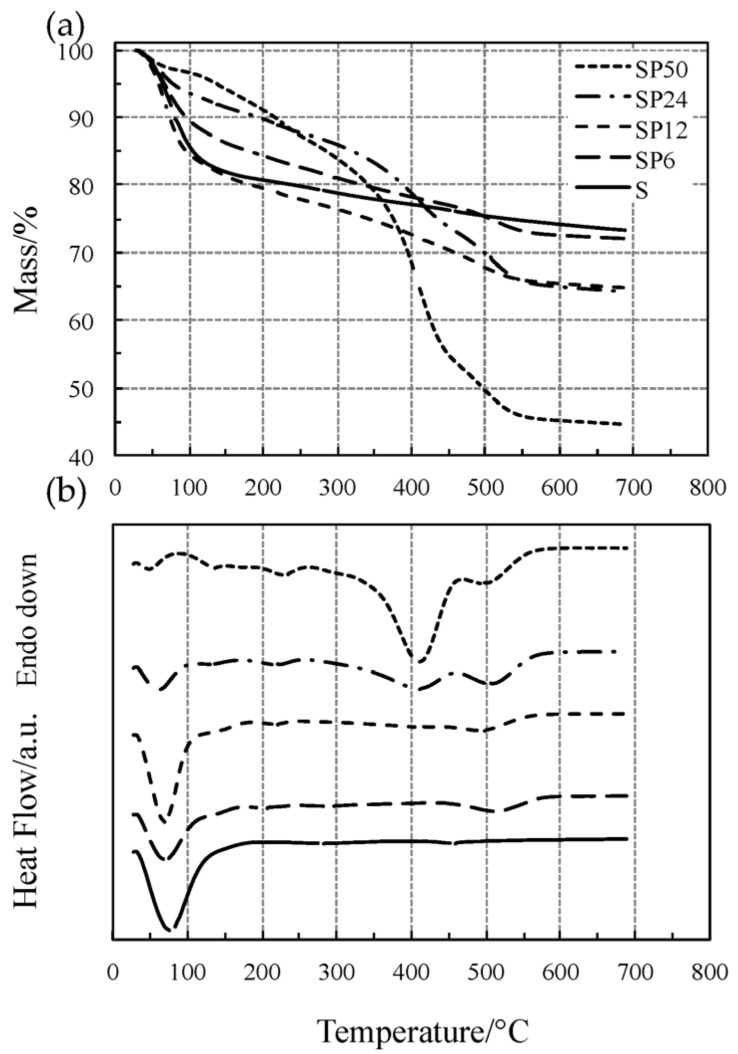
Simultaneous TG (**a**) and DSC (**b**) curves of all the materials tested at 10 °C·min^−1^ in flowing Ar atmosphere.

The TG/DSC curves of pure SiO_2_ (S) and of the SiO_2_/PCL hybrids (SP6, SP12, SP24 and SP50) have been reported in Figure 1.

**Figure 2:**
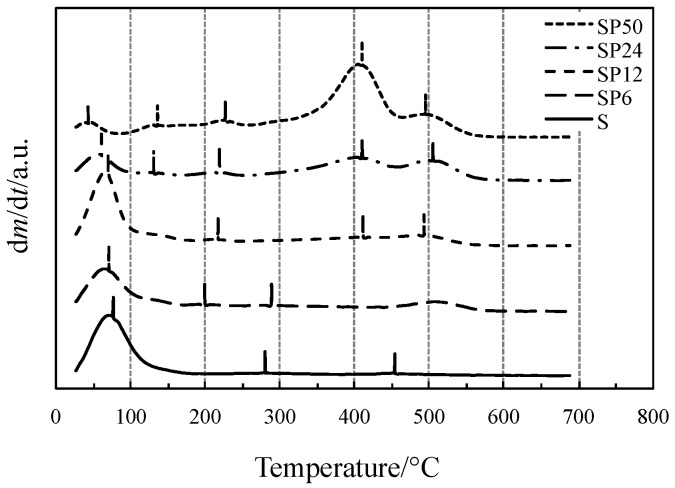
DTG curves of all the materials tested.

Initial and final temperatures of each process accompanied by a mass loss have been more clearly identified by the first-order derivative curves of TG (DTG) curves, displayed in Figure 2.

The TG/DSC curves of all the materials in Figure 1 showed an initial mass loss (corresponding to the first DTG peak) accompanied by an endothermic DSC peak, ascribed to the simultaneous loss of water and alcohol up to 140 °C, except for SP50 (short dotted lines) for which the process ends at around 85 °C. It is clearly evident from Figure 2 (low-temperature region) that the SP hybrid materials, except for SP24 and SP50, show the same thermal behavior as pure S. At temperature higher than 180 °C, dehydration is completed and S undergoes dehydroxylation, elimination of water due to condensation of the hydroxyl surface groups, with a slow and quite constant mass loss rate (linear portion of the TG curve up to 600 °C not detectable by the DTG curve), as found in previous studies [19,20,21,22,24,25]. SP materials (except for SP24 and SP50) show the same thermal behavior up to 300–400 °C, while at higher temperatures, a one- or two-step process took place up to 580–600 °C. This process is accompanied by an endothermic effect, and the intensity of the corresponding DSC peak was found to increase with the amount of PCL in the material, while the degradation temperature shifts towards lower values with an increase in the PCL content.

Similar to what has been observed in a previous study [40], this process is attributable to the thermal degradation of PLC, which usually takes place in two steps of mass loss. On the other hand, the thermal behavior of the PCL-richer materials (SP24 and SP50) is remarkably different from those of the other SP materials. When dehydration is completed at about 85 °C, SP50 undergoes a two-step process up to 260 °C, which can probably be ascribed to dehydroxylation, followed by the two-step thermal degradation of PLC between 300 and 600 °C. The DSC curve recorded two endothermic effects, expressed by two partially convoluted broad peaks: the first one intense up to 455 °C, followed by a second that is a shoulder.

### 2.2. FTIR Evolution Gas Analysis to Provide a Mechanistic Interpretation of the Thermally Stimulated Processes

**Figure 3:**
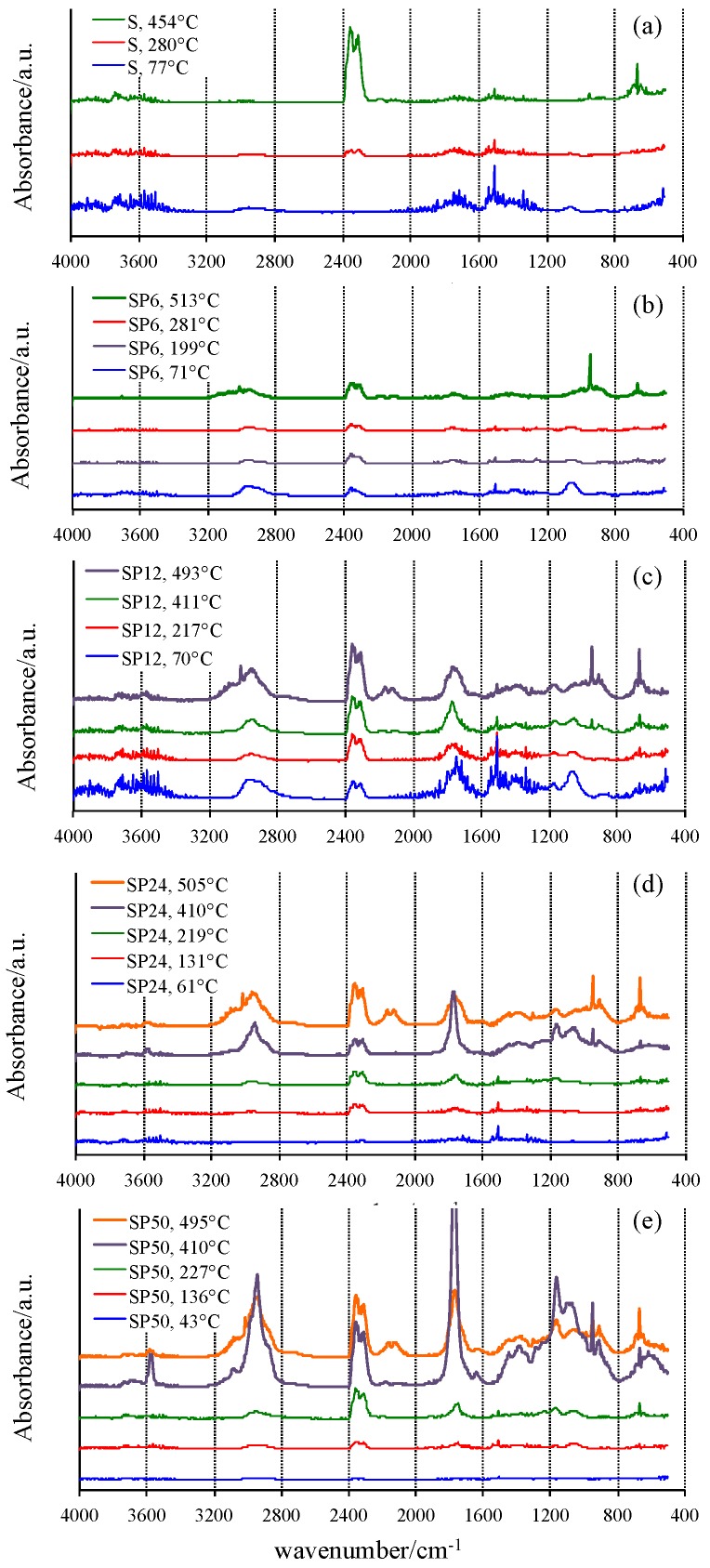
FTIR spectra of the gaseous mixture evolved at selected temperatures from TG experiments.

Vertical bars displayed in Figure 2, close to the DTG peak temperatures where the reaction reaches the maximum rates, represent the temperatures at which the gas or gaseous mixture evolved from TG experiments was collected and sent to the FTIR device. The FTIR spectra of the mixtures collected from the TG/DSC experiments of all materials tested are shown in Figure 3.

A confirmation of the mechanisms hypothesized was found by analyzing the FTIR spectrum of the gases evolved from the samples S during the TG experiment at low temperature (77 °C), showing the typical signals of water. Sharp peaks in the wavenumber regions 4000–3400 cm^−1^ and 2000–1200 cm^−1^ are visible due to the H–O–H stretching and bending vibrations. Moreover, the weak peak at about 1040 cm^−1^ suggests that ethanol [41], used as solvent in the synthesis process and also formed by the hydrolysis reaction that involves the alkoxide precursor tetraethyl orthosilicate (TEOS), is also released in this temperature range from the material in which it was previously embedded in the gel form. A higher release of ethanol was detected in SP6, revealed by the presence of the C–H stretching at 2955 cm^−1^, as well as by the peaks related to the C–C and C–O bonds at 1373, 1249, 1040 and 875 cm^−1^. This can be explained by a decrease of hydrolysis degree and condensation rate caused by the interaction of the –OH groups of the forming inorganic network with the polymer chains in the sol. Therefore, the –OH groups involved in the H–bonds with the C=O of the PCL [26] cannot react with other alkoxide precursors or other oligomers. As a consequence, a higher content of residual ethoxy group is retained in the gel. Moreover, the presence of water and CO_2_ (duplet at 2345–2300 cm^−1^ [41]) is also observed.

Similarly, the amount of ethanol and water released even at low temperature in PCL-rich OIHs (SP12, SP24 and SP50) is higher, due to the higher amount of PCL and, thus, to the higher amount of –OH bonded with it. Moreover, the higher amount of ethanol leads to the formation of a higher amount of CO_2_. The FTIR spectra of pure S and SP6 at 280 and 281 °C, respectively, show that a decrease of the bands attributed to water and ethanol, as well as the development of CO_2_, were observed. SP12 revealed a similar thermal behavior (with respect to those of S and SP6) at low temperatures (69.5 and 217 °C).

At higher temperatures (513 °C for SP6), ethanol is completely degraded, thus leading to the formation of ethylene (as proved by the bands in the following regions: 3300–2900 and 1430 cm^−1^, as well as the sharp band at 950 cm^−1^), CO_2_ and a low amount of CO. The higher amount of ethylene produced from the SP6 sample compared to that of S is due to the higher initial amount of ethanol developed from sample SP6.

FTIR spectra of SP12 at 411 °C showed new bands at 2940, 1770, 1150 and 1050 cm^−1^, which can be ascribable to the formation of caproic acid and ε-caprolactone, both of which are produced from the thermal degradation of PCL, as affirmed by Persenaire and co-workers [40]. Moreover, the bands of CO_2_, CO and the sharp one of ethylene are also visible, even if with low intensity.

SP24 and SP50 showed the same thermal behavior as SP12, but the presence of 5-hexenoic acid in the FTIR spectrum at 410 °C is more evident in the former, while that at 495 °C showed the least decrease; and in the gas phase, ε-caprolactone is mainly present. By increasing the temperature, the band at 3570 cm^−1^, present only in the spectrum of 5-hexenoic acid, decreases. This finding is in agreement with the mechanism of degradation of PCL reported in the literature [40], which is reported to occur in two steps: in the first, the rupture of polyester chains via ester pyrolysis reactions is involved, leading to the formation of 5-hexenoic acid, H_2_O, CO_2_ and a low amount of CO. The second step is attributed to the formation of ε-caprolactone by an unzipping depolymerisation process. Therefore, the intensity of CO_2_ and CO signals is higher in the spectra of those samples compared to those of S and SP6, because when the PCL degrades, CO_2_ and CO also are produced [40,42].

Therefore, the obtained results suggest that in order to obtain OIHs free of internal toxic residual solvents, the materials should be heated at 400 °C.

### 2.3. XRD Analysis to Provide a Mechanistic Interpretation of the Thermally Stimulated Processes

**Figure 4:**
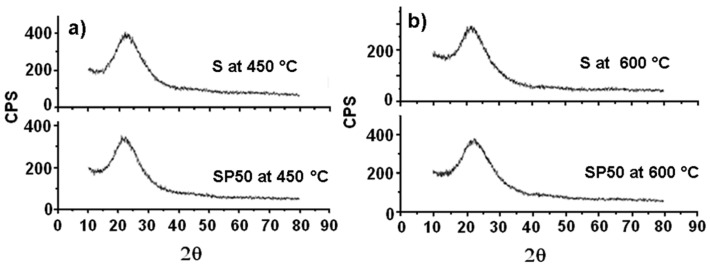
XRD spectra of S and SP50 materials after their treatment at 450 °C (**a**) and 600 °C (**b**).

Figure 4 shows the XRD spectra of both S and SP50 after their thermal treatment at 450 and 600 °C (plots (**a**) and (**b**), respectively). They are all practically amorphous, and only the broad characteristic peak of silica between 15 and 35° is observed [43].

**Figure 5:**
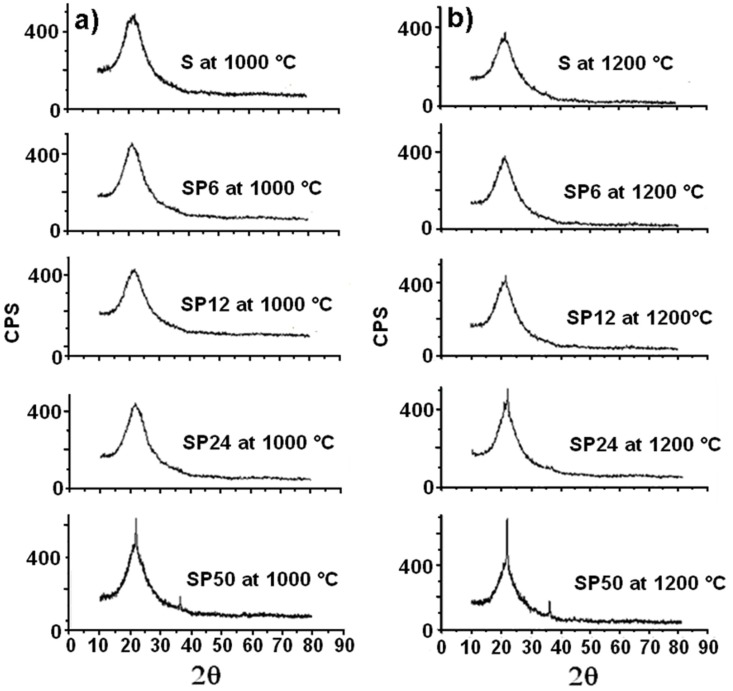
XRD spectra of all the materials tested after their treatment at 1000 °C (**a**) and 1200 °C (**b**).

Furthermore, the S and SP materials are revealed to be amorphous, even after their treatment at 1000 °C, as is clearly evident from the XRD spectra in Figure 5a.

SP50 shows an initial crystalline structure (that of β-cristobalite, a high-temperature stable polymorph of silica). Crystallization of β-cristobalite seems to occur more evidently (especially in the case of SP50) in all the materials (even in pure S) after their treatment at 1200 °C, as the XRD spectra in Figure 5b show clearly. This result partly confirmed what was obtained in a previous study [44], where amorphous silica crystallized in cristobalite at 1000 °C due to a local rearrangement of the amorphous material (similar to β-cristobalite). The explanation for this is that the instantaneous local atomic arrangement of amorphous SiO_2_ is similar to that of β-cristobalite [45]. Usually, a phase change transformation from quartz to β-cristobalite only takes place when the temperature is about 1470 °C [38]. Then, at high temperature, it is easier to observe the crystallization of amorphous SiO_2_ into β-cristobalite than the phase change from quartz.

## 3. Materials and Methods

### 3.1. Synthesis of the Hybrid Materials

The SiO_2_/PCL hybrid materials were synthesized by means of the sol-gel method, according to a procedure reported in detail in Catauro et al. [26]. The PCL-free SiO_2_ was obtained from a Silica sol prepared by adding drop by drop water to a solution of the alkoxide precursor tetraethyl orthosilicate (TEOS, sigma Aldrich, Milan, Italy) in pure ethanol (EtOH, 99.8%, Sigma Aldrich, Milan, Italy) and nitric acid (65%, Sigma Aldrich, Milan, Italy). The last was used as catalyst of the hydrolysis reaction, which involves the alkoxide precursor. In the sol, the following molar ratios between the reagents are achieved: H_2_O/TEOS = 2; EtOH/TEOS = 6; TEOS/HNO_3_ = 1.7.

To synthesize the hybrid materials, different amounts of PCL (Sigma Aldrich, Milan, Italy), with an average molar mass of 10.0 kg·mol^−1^, were dissolved in chloroform (Sigma Aldrich, Milan, Italy) and then added to the silica sol. All reagents were used as received, without further purification. Finally, five materials were synthesized: pure SiO_2_ and four SiO_2_/PCL hybrids containing 6, 12, 24 and 50 wt % of PCL.

### 3.2. Instrumental Details to Study the Thermal Behavior of the Hybrid Materials

After their preparation, all the materials investigated were gently ground in an agate mortar for some minutes to reduce them into fine powders. Then, samples of the obtained powders were further characterized by coupled TG/Differential scanning calorimetry (TG/DSC), coupled thermogravimetry/Fourier transform infrared spectroscopy (TG/FTIR) and X-ray diffraction (XRD) analyses. The thermal behavior of the OIHs was studied under an inert nitrogen flowing atmosphere (60 mL·min^−1^) up to 700 °C at a heating rate of 10 °C·min^−1^ using a simultaneous Mettler Toledo TG/DSC 2950 instrument （Mettler Toledo, Columbus, OH, USA), equipped with a STARe software (version 12.00, Mettler Toledo, Columbus, OH, USA). The instrument was equipped with two identical cylindrical crucibles, one for the reference filled with alumina in powder form and one for the sample, uniformly covered with about 20–25 mg of solid to uniformly cover the bottom surface area of the crucible. Calibration of the sample temperature was performed using very pure indium and zinc reference materials (purity higher than 99.998%), thus assuming a final average uncertainty *u*(*T*) = ±1 K was estimated over the whole temperature range.

In order to collect more information to support a reasonable mechanism associated to the thermally stimulated processes that take place in the OIHs submitted to the TG/DSC experiments, the TG/FTIR experiments were performed using a SETARAM 92-16.18 TG apparatus (SETARAM, Caluire, France) under a stream of argon of 40 mL min^−1^ in the temperature range between 25 and 700 °C at 10 °C min^−1^. This instrument has been equipped with 250 µL alumina crucibles filled with about 100–150 mg of sample to obtain the minimum amount of gaseous species required for FTIR measurements. A preliminary blank experiment was performed using empty crucibles under identical experimental conditions of the samples tested. All the experimental data were collected and analyzed using the Calisto software (version 1.38, SETARAM, Caluire-et-Cuire, France). The vapors evolved during the TG experiments were conveyed to a Thermofisher Scientific Nicolet iS10 Spectrophotometer (Thermofisher, Waltham, MA, USA) linked through a heated transfer line kept at 200 °C. The instrument allows monitoring the actual reaction trend by collecting a spectrum each 11 s, being eight scans performed at 0.5 cm^−1^ intervals with a resolution of 4 cm^−1^.

The thermal treatment of the sample powders was carried out in a muffle furnace for 2 h under argon purge gas atmosphere at 450, 600, and 1000 °C (temperatures selected on the basis of a careful examination of their thermal behavior shown by the TG experiments). In order to verify the occurrence of crystallization processes at higher temperatures a supplementary thermal treatment at 1200 °C was also performed in a tubular oven under argon flowing atmosphere for 90 min.

All the crystalline phases were identified by XRD analysis using a Philips diffractometer equipped with a PW 1830 generator （Philips, Amsterdam, The Netherlands）, where the source of X-ray is given by a Cu-Kα radiation (λ = 0.15418 nm).

## 4. Conclusions

The thermal behavior of four SiO_2_-PCL hybrid materials containing increasing amount of poly(ε-caprolactone) (PCL) has been studied by simultaneous TG/DSC and compared with that of pure SiO_2_. The FTIR analysis of the gases evolved at defined temperatures from the samples submitted to TG experiments enabled identification of the mechanisms of dehydration, ethanol release (at low temperature) and PCL thermal degradation, occurring upon heating. In particular, the thermal degradation of PCL in SP50 leads to 5-hexenoic acid, H_2_O, CO_2_, CO and ε-caprolactone. After the samples’ treatment at 450, 600 and 1000 °C, the X-ray diffraction (XRD) spectra revealed that they were still amorphous, while the presence of cristobalite was found in the richest-PCL material.

The knowledge of the thermal behavior of those bioactive and biocompatible sol-gel hybrid materials, as well as of the degradation processes that take place within them upon heating, has a key role in the development of new performing biomaterials. In fact, this information allows modulation of the microstructure of the obtained biomaterials and, thus, their biological properties. Therefore, the correlation between the obtained results and further information about both structural modifications induced by heating and biological performance as a function of the heat treatment could be useful in the near future for the design of biomaterials suitable for specific clinical applications.
